# Cytochrome *b*_5_ reductase 3 overexpression and dietary nicotinamide riboside supplementation promote distinctive mitochondrial alterations in distal convoluted tubules of mouse kidneys during aging

**DOI:** 10.1111/acel.14273

**Published:** 2024-07-12

**Authors:** M. Pérez‐Rodríguez, A. García‐Verdugo, L. M. Sánchez‐Mendoza, A. Muñoz‐Martín, N. Bolaños, C. Pérez‐Sánchez, J. A. Moreno, M. I. Burón, R. de Cabo, J. A. González‐Reyes, J. M. Villalba

**Affiliations:** ^1^ Departamento de Biología Celular, Fisiología e Inmunología Universidad de Córdoba, Campus de Excelencia Internacional Agroalimentario Córdoba Spain; ^2^ Experimental Gerontology Section, Translational Gerontology Branch National Institute on Aging, National Institutes of Health Baltimore Maryland USA; ^3^ Maimonides Biomedical Research Institute of Cordoba (IMIBIC), Hospital Universitario Reina Sofía Córdoba Spain

**Keywords:** aging, cytochrome *b*_5_ reductase 3, distal convoluted tubules, mitochondria, mitochondria‐endoplasmic reticulum contact sites, nicotinamide riboside, quantitative transmission electron microscopy, transcriptomics

## Abstract

The kidney undergoes structural and physiological changes with age, predominantly studied in glomeruli and proximal tubules. However, limited knowledge is available about the impact of aging and anti‐aging interventions on distal tubules. In this study, we investigated the effects of cytochrome *b*_5_ reductase 3 (CYB5R3) overexpression and/or dietary nicotinamide riboside (NR) supplementation on distal tubule mitochondria. Initially, transcriptomic data were analyzed to evaluate key genes related with distal tubules, CYB5R3, and NAD^+^ metabolism, showing significant differences between males and females in adult and old mice. Subsequently, our emphasis focused on assessing how these interventions, that have demonstrated the anti‐aging potential, influenced structural parameters of distal tubule mitochondria, such as morphology and mass, as well as abundance, distance, and length of mitochondria‐endoplasmic reticulum contact sites, employing an electron microscopy approach. Our findings indicate that both interventions have differential effects depending on the age and sex of the mice. Aging resulted in an increase in mitochondrial size and a decrease in mitochondrial abundance in males, while a reduction in abundance, size, and mitochondrial mass was observed in old females when compared with their adult counterparts. Combining both the interventions, CYB5R3 overexpression and dietary NR supplementation mitigated age‐related changes; however, these effects were mainly accounted by NR in males and by transgenesis in females. In conclusion, the influence of CYB5R3 overexpression and dietary NR supplementation on distal tubule mitochondria depends on sex, genotype, and diet. This underscores the importance of incorporating these variables in subsequent studies to comprehensively address the multifaceted aspects of aging.

## Graphical abstract

The effect of cytochrome *b*_5_ reductase 3 overexpression and nicotinamide riboside supplementation on kidney distal convoluted tubules was analyzed through a transcriptomic and electron microscopy approach. The results indicated that the combination of both interventions prevented aging‐triggered alterations on key mitochondrial population features, such as mitochondrial morphology and mass, and mitochondria‐endoplasmic reticulum contact sites. Notably, the effects were predominantly associated with dietary supplementation in males, while being more closely linked to cytochrome *b*_5_ reductase 3 overexpression in females.

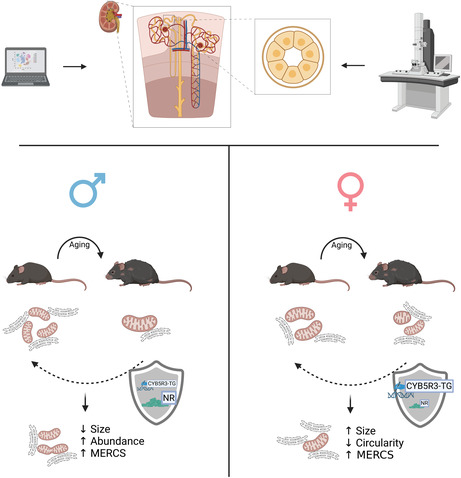

**Abbreviations**
a/A: relative mitochondrial mass
AF: adult female
AM: adult male
Ank2: Ankyrin 2
Atp2b4: ATPase Plasma Membrane Ca^2+^ Transporting 4
Calb1: Calbindin 1
Clcnkb: Chloride Voltage‐Gated Channel Kb
CR: caloric restriction
CT: control
Cyb5a: Cytochrome B5 Type A
Cyb5b: Cytochrome B5 Type B
Cyb5r3: Cytochrome B5 Reductase 3
CYB5R3: Cytochrome *b*_5_ reductase 3
DCT: distal convoluted tubule
DEG: differentially expressed genes
FDR: false discovery rate
GEO: Gene Expression Omnibus
GO: Gene Ontology
Ido2: Indoleamine 2,3‐Dioxygenase 2
Kcnj1: Potassium Inwardly Rectifying Channel Subfamily J Member 1
Kcnj10: Potassium Inwardly Rectifying Channel Subfamily J Member 10
Kl: Klotho
Klhl3: Kelch Like Family Member 3
MERCS: mitochondria‐endoplasmic reticulum contact sites
Na: relative mitochondrial abundance
Nampt: Nicotinamide Phosphoribosyltransferase
Nmrk1: Nicotinamide Riboside Kinase 1
NR: nicotinamide riboside
OF: old female
OM: old male
PCT: proximal convoluted tubule
Qprt: Quinolinate Phosphoribosyltransferase
SEM: standard error of the mean
Slc12a3: Solute Carrier Family 12 Member 3
Slc8a1: Solute Carrier Family 8 Member A1
Suox: Sulfite Oxidase
TEM: transmission electron microscopy
TG: transgenic
Trpm6: Transient Receptor Potential Cation Channel Subfamily M Member 6
Wnk1: WNK Lysine Deficient Protein Kinase 1
WT: wild‐type

## 1 INTRODUCTION

Elucidating how humans can preserve health while extending their longevity is one of the greatest current scientific challenges, and multiple experimental approaches have been developed to achieve this goal in model organisms (Mitchell et al., 2015). Caloric restriction (CR) has been established as the quintessential nutritional intervention able to postpone the onset of disease, extend lifespan, and improve the quality of life (Mitchell et al., 2015). On the other hand, dietary supplementation with resveratrol, metformin, rapamycin, or nicotinamide riboside (NR) also attenuates hallmarks of aging and, in some cases, promote life‐ and/or health‐span extension in animal models (Madeo et al., 2019). For instance, supplementation with NR, a precursor of NAD^+^ synthesis, induces improvements in mitochondrial status via increasing NAD^+^ levels and NAD^+^/NADH ratio in the cell (Chini et al., 2024). Moreover, genetic interventions via overexpression of NADH‐cytochrome *b*_5_ reductase‐3 (CYB5R3), an enzyme that participates in several key metabolic processes and increases NAD^+^/NADH ratio as well, has been also shown to improve both health and lifespan in transgenic mice (Martin‐Montalvo et al., 2016).

Like other organs, the kidney undergoes physiological and morphological changes with age. These changes include glomerular sclerosis, thickening of the glomerular basement membrane, and foot processes effacement (Fang et al., 2020). We reported previously that deleterious changes were less pronounced in male mice fed a CR diet. Additionally, we also described significant changes in mitochondrial morphology and mass in proximal convoluted tubule (PCT) cells and documented that the positive impact of CR was influenced by the predominant type of dietary fat, underscoring the significance of diet in preserving subcellular architecture during the aging process (Calvo‐Rubio et al., 2016).

Surprisingly, the distal convoluted tubule (DCT) has been relatively overlooked in the context of aging, despite its important role in the homeostasis of Na^+^, K^+^, Cl^−^, divalent cations, and, by extension, blood pressure (Subramanya & Ellison, 2014). Furthermore, the anti‐aging Klotho protein is mainly expressed in DCT cells (Buchanan et al., 2020). Reduced Klotho activity with age leads to heightened intracellular phosphate and increased cellular senescence, resulting in ROS‐induced damage and diminished autophagy in old mice (Buchanan et al., 2020).

These data suggest that DCT may be an interesting target in the study of aging. Specifically, little is known about the effect of CYB5R3 overexpression or supplementation with biosynthetic precursors of NAD^+^ synthesis in this segment of the nephron. On the other hand, DCT cells are rich in mitochondria (McCormick & Ellison, 2015); therefore, they constitute an excellent site to investigate possible changes in the morphology and mass of this organelle with age and anti‐aging interventions.

Production and accumulation of reactive oxygen species (ROS) causes damage to tissues, contributing critically to the aging of the individual (Barja, 2013). Being the primary source of ROS, mitochondria play a central role in aging. Beyond ROS‐induced damage, impaired mitochondrial physiology/morphology and disabled mitophagy also emerge as important hallmarks of this process (López‐Otín et al., 2023). Among others, mitochondria‐endoplasmic reticulum contact sites (MERCS) have been also associated with these crucial events since they are involved in redox signaling regulation, mitochondrial DNA replication, mitochondrial dynamics, and autophagy (Moltedo et al., 2019). Considering the significance of the NAD^+^/mitophagy axis (Aman et al., 2020), and the relationship between NAD^+^ and DNA repair or mitochondrial maintenance (Croteau et al., 2017), it might be interesting to explore MERCS changes induced by interventions aimed at increasing NAD^+^ levels, such as CYB5R3 overexpression and NR supplementation.

Access to high‐quality data is a crucial step to comprehensively explore potential underlying changes and mechanisms. Utilizing available high‐throughput data and employing bioinformatic tools can yield valuable insights (Edgar et al., 2002). Additionally, electron microscopy stands out as the gold standard for analyzing mitochondrial morphology and mass (Pérez‐Rodríguez et al., 2023), and is an ideal technology for studying MERCS as well.

The aim of this paper is to investigate whether the overexpression of CYB5R3 and/or dietary supplementation with NR induce changes in mitochondrial morphology and mass, as well as in MERCS features, of DCT cells during aging. We further seek to elucidate whether CYB5R3 overexpression along with NR supplementation presents synergistic effects on these DCT components. Since both longevity in mammals and the outcomes to anti‐aging interventions are influenced by sex, we have incorporated both male and female mice into our study.

## 2 MATERIALS AND METHODS

### 2.1 Transcriptomic analysis using public datasets

The independent dataset with accession number GSE175854 (Han et al., 2021), publicly available in the database Gene Expression Omnibus (GEO; Edgar et al., 2002), was analyzed. It comprises total ribosome‐depleted RNA sequencing data from kidneys of 8 C57BL/6J mice. The samples were stratified by sex (male and female) and age (adults –16 weeks old– and olds –86 weeks old–). Subsequent analysis included two adult males (AM), two old males (OM), two adult females (AF), and two old females (OF).

Data analysis was performed using the R software through RStudio (Rstudio Team, 2020). Differentially Expressed Genes (DEG) were obtained using EdgeR (Robinson et al., 2010). First, genes with low expression levels, indicated by counts below 10, were removed. Then, Trimmed Mean of M‐values method was used for data normalization. After data exploration to identify potential outlier samples and estimate dispersion, DEG analysis was carried out. DEG analysis was performed using a Generalized Linear Model approach and the Likelihood Ratio Test.

Here, multiple comparisons were considered. The males versus females comparison was examined to obtain the main differences between the sexes. Old versus adult comparison was conducted to determine the main effect of age. The interaction sex x age was also explored. Next, AM versus OM, AF versus OF, AM versus AF, and OM versus OF comparisons were also analyzed to obtain differences with age in males and females, and between adults and older specimens, respectively. The Benjamini–Hochberg procedure (Benjamini & Hochberg, 1995) was applied for *p*‐value adjustment to control the false discovery rate (FDR). FDR < 0.05 was set as the threshold for statistical significance, and FDR < 0.1 to indicate trends.

Finally, genes related to DCT, CYB5R3, and NAD^+^ biosynthesis were identified, focusing on those exhibiting differential expression across the comparisons. DCT‐related genes were defined based on a published mouse kidney atlas (Novella‐Rausell et al., 2023). The top 50 meta‐markers for DCT cells and top 50 for DCT‐like DCT‐CNT cells were extracted, and a list of 82 unique DCT‐related genes was obtained (see, Supplementary List S1). For the selection of CYB5R3‐related genes, STRING platform was used (Szklarczyk et al., 2023). A network with CYB5R3 and its five most confident interactors was generated to create a list of six CYB5R3‐related genes (Supplementary List S2). The list of NAD^+^ biosynthesis‐related genes was obtained from Xie et al. (2020) where a total of 12 key genes were selected (Supplementary List S3). Subsequently, DEGs that were included in those lists were identified, and an enrichment analysis was carried out using STRING.

Differentially expressed CYB5R3, and NAD^+^ biosynthesis‐related genes, as well as genes associated with the enriched terms derived from differentially expressed DCT‐related genes, were represented in graphs, including normalized count means, standard error of the mean (SEM), and statistical significance, using GraphPad Prism 8 (GraphPad Software Inc., San Diego, CA, USA).

## 3 ANIMALS AND DIETS: IN VIVO MODEL FOR ELECTRON MICROSCOPY

In this study, we used male and female mice of wild‐type (WT) or CYB5R3‐overexpressing (transgenic, TG) genotypes in a C57BL/6J genetic background. TG mice were generated by introducing the rat CYB5R3 gene as described in Martin‐Montalvo et al. (2016) and maintained at the Servicio de Animales de Experimentación (SAEX) at the University of Córdoba (Spain).

Founder pairs were established by crossing TG males with WT females of the same genetic background, obtained from Charles River (Barcelona, Spain). Subsequently, the resulting litters were identified by PCR genotyping using genomic DNA obtained from ear tissue.

The mice were housed in sterile cages with filter lids in a controlled environment with a 12‐h light/dark cycle at 22°C and free access to water. They were fed a standard chow diet from weaning until they reached 3 months of age. Then, animals were fed an AIM93M diet either supplemented or not (Control; CT) with NR (NIAGEN®, ChromaDex, Los Angeles, CA, USA) at a dose of 400 mg per kilogram of animal weight per day. The mice were maintained under these conditions for 4 or 21 months, reaching 7 months (adult; A) and 24 months (old; O) of age at the time of sacrifice, respectively. In this study, 16 experimental groups were established according to age, sex, CYB5R3 genotype, and dietary NR supplementation.

After the intervention period, the animals were euthanized by cervical dislocation under isoflurane anesthesia, and tissue samples were taken. All procedures involving animals were approved by the Bioethics and Biosafety Committee of the University of Córdoba (Spain) and were authorized by the Department of Agriculture, Fisheries, and Rural Development of the Andalusian Government (Spain; authorization code: 06/06/2019/098).

## 4 PROCESSING OF SAMPLES FOR TRANSMISSION ELECTRON MICROSCOPY (TEM)

A conventional electron microscopy sample preparation protocol was performed (see Data S1 for a detailed protocol). First, kidney samples were fixed in glutaraldehyde in sodium cacodylate buffer. Then, samples were dehydrated in a total ascendent ethanol series, transferred to propylene and sequentially infiltrated in EMbed 812 resin (EMS, USA). Afterwards, samples blocks were formed in silicon molds. After trimming the blocks, ultra‐thin sections (60–80 nm thick) were obtained for studies with electron microscope. Sections were obtained using a Reichert‐Jung ultramicrotome (Germany) and a diamond knife (Diatome, Switzerland), mounted on nickel grids and contrasted with uranyl acetate and lead citrate (EMS, USA).

Ultra‐thin sections were observed and photographed using a JEOL JEM 1400 transmission electron microscope. Images of whole DCT cells were taken at 8000 X for mitochondrial and MERCS analysis. A set of selection criteria was established for capturing photographs of DCT cells, which included well‐defined cell boundaries, prominent central or apical nuclei, absence of microvilli at the apical cell border (tubular lumen), and abundant folds of the plasma membrane at the basal cell dominium. These criteria are consistent with those described in McCormick and Ellison (2015) and Delaney et al. (2018).

## 5 QUANTITATIVE ELECTRON MICROSCOPY ANALYSIS

Planimetric analysis of mitochondria and MERCS was performed using ImageJ 1.53 K software (N.I.H., USA), following previously described methods (Neikirk et al., 2023). After setting the appropriate scale, a tool in the program was used to delineate and mark the contours of the plasma membrane (complete cell), nuclei, mitochondrial profiles, and MERCS descriptors contained in each picture. Afterwards, data on the previously chosen parameters were collected.

The morphometric parameters studied were of two types: planimetric and “abundance” or mass. The planimetric parameters included mitochondrial area and circularity. For mass study, “mitochondrial relative mass” (a/A) and “mitochondrial relative abundance” (Na) were used. The former was obtained by summing the areas of all mitochondria within a cell and dividing it by the cytoplasmic area of that cell. The second parameter was obtained by dividing the number of mitochondria by the cytoplasmic area. The cytoplasmic area of each cell was obtained by subtracting the nucleus from the complete cell area. A MERCS was scored when a juxtaposition of mitochondria with an endoplasmic reticulum cisterna appeared at a distance ≤80 nm (see Moltedo et al., 2019 and the references cited therein). MERCS distance was determined as the thickness of the contact zone, and MERCS length as the distance along which the MERCS distance remained constant (Cardoen et al., 2024). Relative MERCS abundance was obtained by dividing the number of MERCS by the number of mitochondria within each cell. Table S1 shows the number of animals, cells, mitochondria, and MERCS measured in each experimental group.

Data obtained from the ImageJ software were recorded in a spreadsheet, where average values per cell were calculated for subsequent analysis. These data were then imported into the statistical software GraphPad Prism 8. Continuous variables, including mitochondrial area, circularity, a/A, and Na, as well as MERCS distance, length and abundance were initially entered, and organized based on the independent variables: sex, age, genotype, and diet. First, outliers were detected and removed from the analysis. Subsequently, the normality and homogeneity of variances were assessed for each combination of groups within the three independent variables. Finally, statistical tests were conducted. Thus, two distinct three‐way ANOVA configurations were performed: one involving age, genotype, and diet as independent factors for males and females, and another involving sex, genotype, and diet for adult and old animals. After identifying significant alterations in the three‐way ANOVA, subsequent two‐way ANOVA analyses were conducted for each subset derived from the three‐way ANOVA (adult males, old males, adult females, or old females). All statistical analyses were carried out with a significance threshold set at a *p* < 0.05, additionally using a *p* < 0.1 to indicate trends. Post hoc tests were conducted using the Sidak test for multiple comparisons in cases of significant ANOVA results. Sidak's test results from the three‐way ANOVA data were used to discern specific distinctions related to age or sex. Similarly, Sidak's test results from the two‐way ANOVA were employed to compare among multiple groups within the two‐way ANOVA. The results were represented in bar graphs as means, including SEM and statistical significance. Tables S2 and S3 show the mean ± SEM values for each experimental group of the mitochondrial and MERCS parameters, respectively.

## 6 RESULTS

### 6.1 Distinctive transcriptomic patterns associated with sex and age

The transcriptomic study revealed sex, and age‐dependent variations across several genes related to DCT, CYB5R3, and NAD^+^ biosynthesis as described below.

### 6.2 DCT‐related genes

**FIGURE 1:**
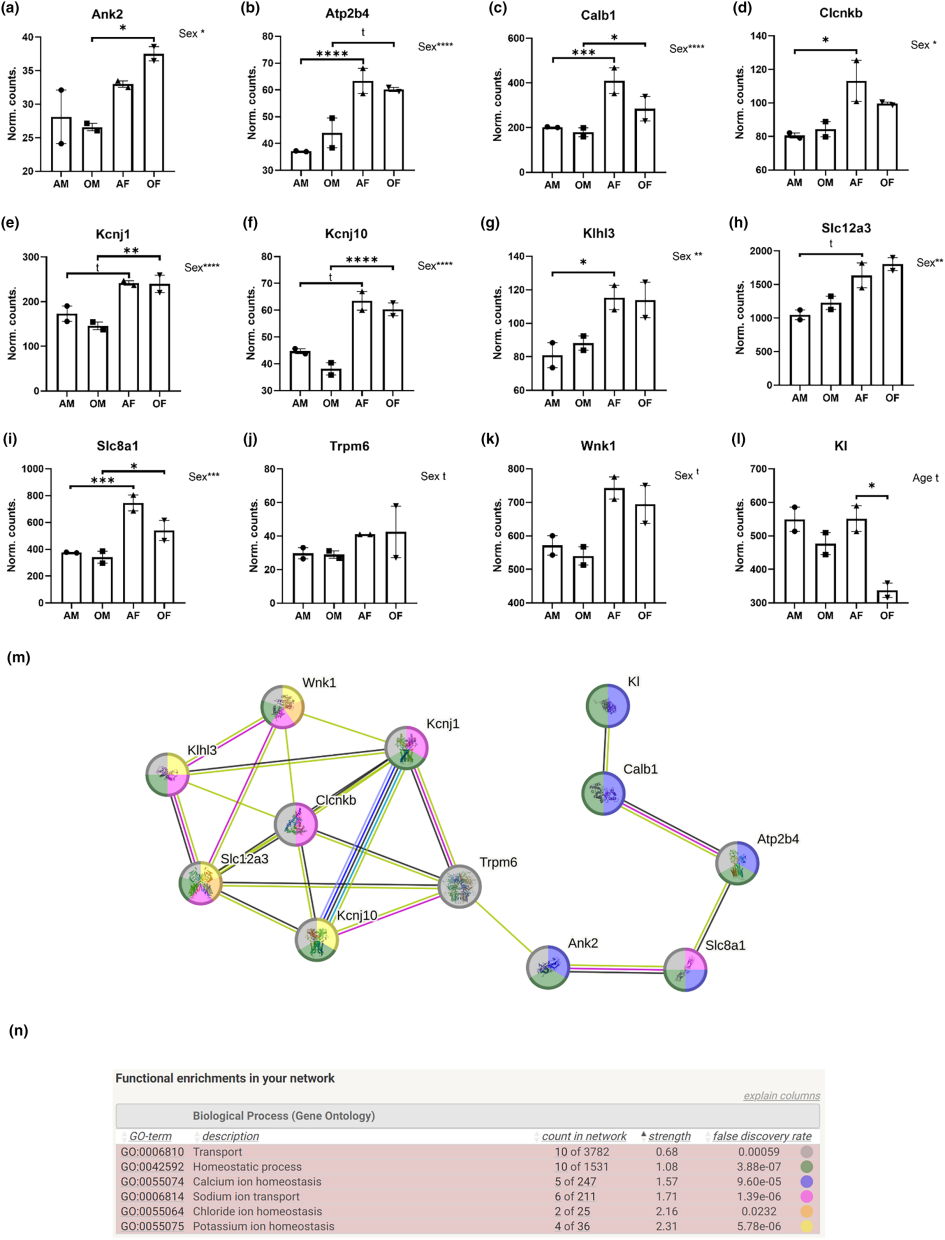
DCT‐related genes displaying differential expression and enriched GO terms. (a–l) Show mean and SEM values of the displayed DCT‐related genes. (m) Depicts the network generated by STRING. (n) Displays enriched GO terms in the network and refers to the color code of (m). Sex: Males versus females. Age: Adult versus old. AF, adult females; AM, adult males; OF, old females; OM, old males; t, tendency (FDR < 0.1); *FDR < 0.05; **FDR < 0.01; ***FDR < 0.001; ****FDR < 0.0001.

Out of the 82 meta‐markers selected as DCT‐related genes, 53 manifested distinctive patterns of expression. Each of these 53 genes exhibited statistically significant differential expression in at least one of the designated comparisons. Among these 53 genes, 17 were integrated into a functional protein association network using STRING (see Figure S1). Enrichment analysis identified 12 genes linked to enriched Gene Ontology (GO) terms associated with biological processes, such as transport, homeostatic processes, calcium ion homeostasis, sodium ion transport, chloride ion homeostasis, and potassium ion homeostasis (Figure 1m,n). The expression profiles of these 12 genes were graphically depicted for the four experimental conditions (Figure 1a–l).

Ank2, Atp2b4, Calb1, Clcnkb, Kcnj1, Kcnj10, Klhl3, Slc12a3, Slc8a1, Trpm6, and Wnk1 exhibited sex differences, with transcript levels higher in females than males (Figure 1a–k, FDR from <0.1 to <0.0001). However, these differences did not show the same level of significance when comparing adult and old animals separately. Atp2b4, Calb1, Clcnkb, Klhl3, Slc12a3, and Slc8a1 exhibited clearer differences among adult animals (Figure 1b–d,g–i), whereas Ank2, Kcnj1, and Kcnj10 showed them in aged animals (Figure 1a,e–f). On the other hand, Kl exhibited a tendency to decrease with age regardless of sex (Figure 1l, FDR < 0.1). Nevertheless, this effect was more pronounced and reached statistical significance in females (Figure 1l, FDR < 0.05).

### 6.3 CYB5R3‐related genes

**FIGURE 2:**
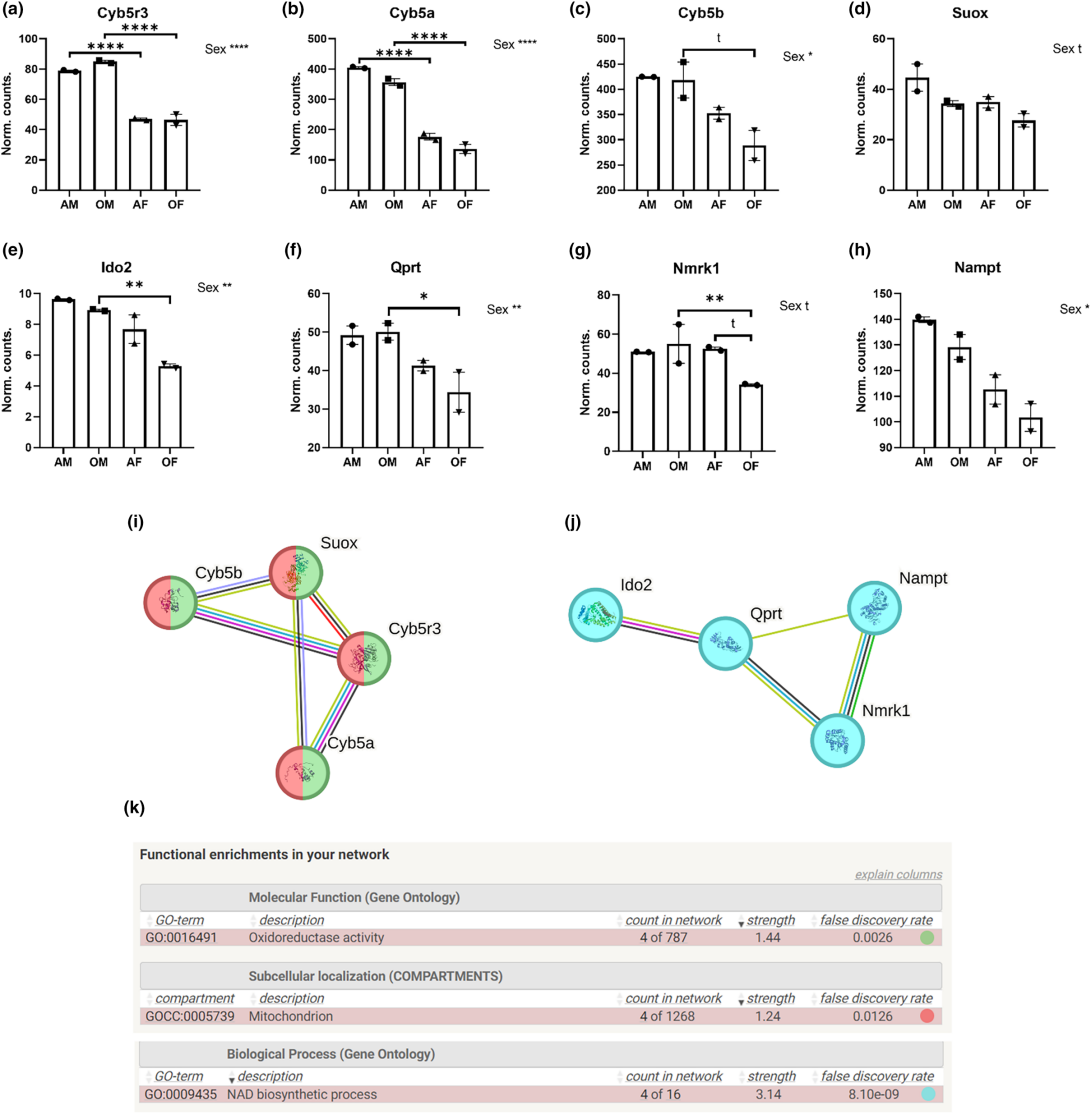
CYB5R3 and NAD^+^ biosynthesis‐related genes displaying differential expression and enriched terms. (a–d) Show mean and SEM values of the displayed CYB5R3‐related genes. (e–h) Show mean and SEM values of the displayed NAD^+^ biosynthesis‐related genes. (i) Depicts the network generated by STRING using displayed CYB5R3‐related genes. (j) Depicts the network generated by STRING using displayed NAD^+^ biosynthesis‐related genes. (k) Displays enriched terms in the networks above and refers to the color code of (i, j). Sex: Males versus females. Age: Adult versus old. AF, adult females; AM, adult males; OF, old females; OM, old males; t, tendency (FDR < 0.1); *FDR < 0.05; **FDR < 0.01; ***FDR < 0.001; ****FDR < 0.0001.

Significant differences between sexes were observed in 4 out of the 6 analyzed CYB5R3‐related genes: Cyb5r3, Cyb5a, Cyb5b, and Suox (see Figure 2a–d). These genes were associated with oxido‐reduction processes and the mitochondria (Figure 2i,k). Cyb5r3 and Cyb5a displayed a very similar expression profile, with transcript levels being higher in males than females (Figure 2a,b, FDR < 0.0001). These differences were also evident when comparing adult animals of both sexes on one hand and aged animals of both sexes on the other (Figure 2a,b, FDR < 0.0001). Cyb5b also demonstrated sex differences (Figure 2c, FDR < 0.05), although this difference was more pronounced in aged animals (Figure 2c, FDR < 0.1). Similarly, Suox showed a trend towards lower levels in female animals (Figure 2d, FDR <0.1).

### 6.4 NAD ^+^ biosynthesis‐related genes

Among the genes associated with NAD^+^ biosynthesis, 4 of them showed differential expression (Figure 2e–h). Ido2 and Qprt are involved in the NAD^+^ de novo synthesis pathway, whereas Nmrk1 and Nampt are part of the salvage pathway (Figure 2j,k). Ido2 and Qprt exhibited similar expression profiles, with transcript levels being higher in males than females for both genes (Figure 2e,f, FDR < 0.01). However, these differences were more pronounced when comparing aged animals (Figure 2e, FDR < 0.01, and Figure 2f, FDR < 0.05). Nmrk1 showed a trend towards sex differences (Figure 2g, FDR < 0.1). In females, aging tended to decrease Nmrk1 levels (Figure 2g, FDR < 0.1), while in males, no apparent differences were observed. Thus, Nmrk1 levels were found to be higher in old males than in old females (Figure 2g, FDR < 0.01). Nampt also exhibited sex differences, with their levels being again higher in males than in females (Figure 2h, FDR < 0.05).

## 7 ELECTRON MICROSCOPY ANALYSIS

**FIGURE 3:**
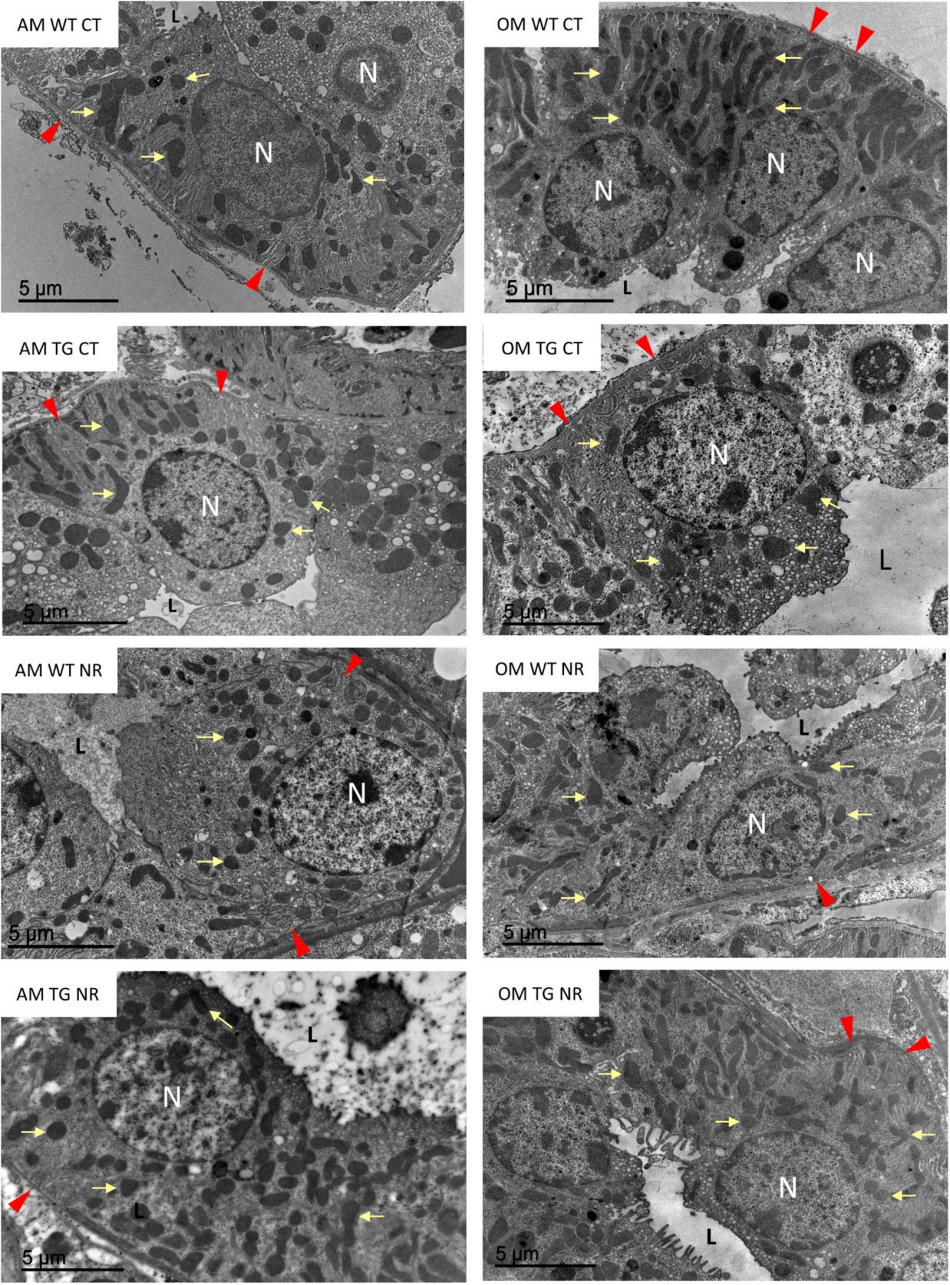
The examples of distal convoluted tubule epithelial cells from male mice from the different experimental groups. The panels on the left display images of adult mice, whereas those on the right correspond to old animals. Each panel includes a tag indicating the experimental group. Some examples of mitochondria are marked with yellow arrows while red arrowheads indicate basal folds of the plasma membrane. L, tubular lumen; N, nucleus.

**FIGURE 4:**
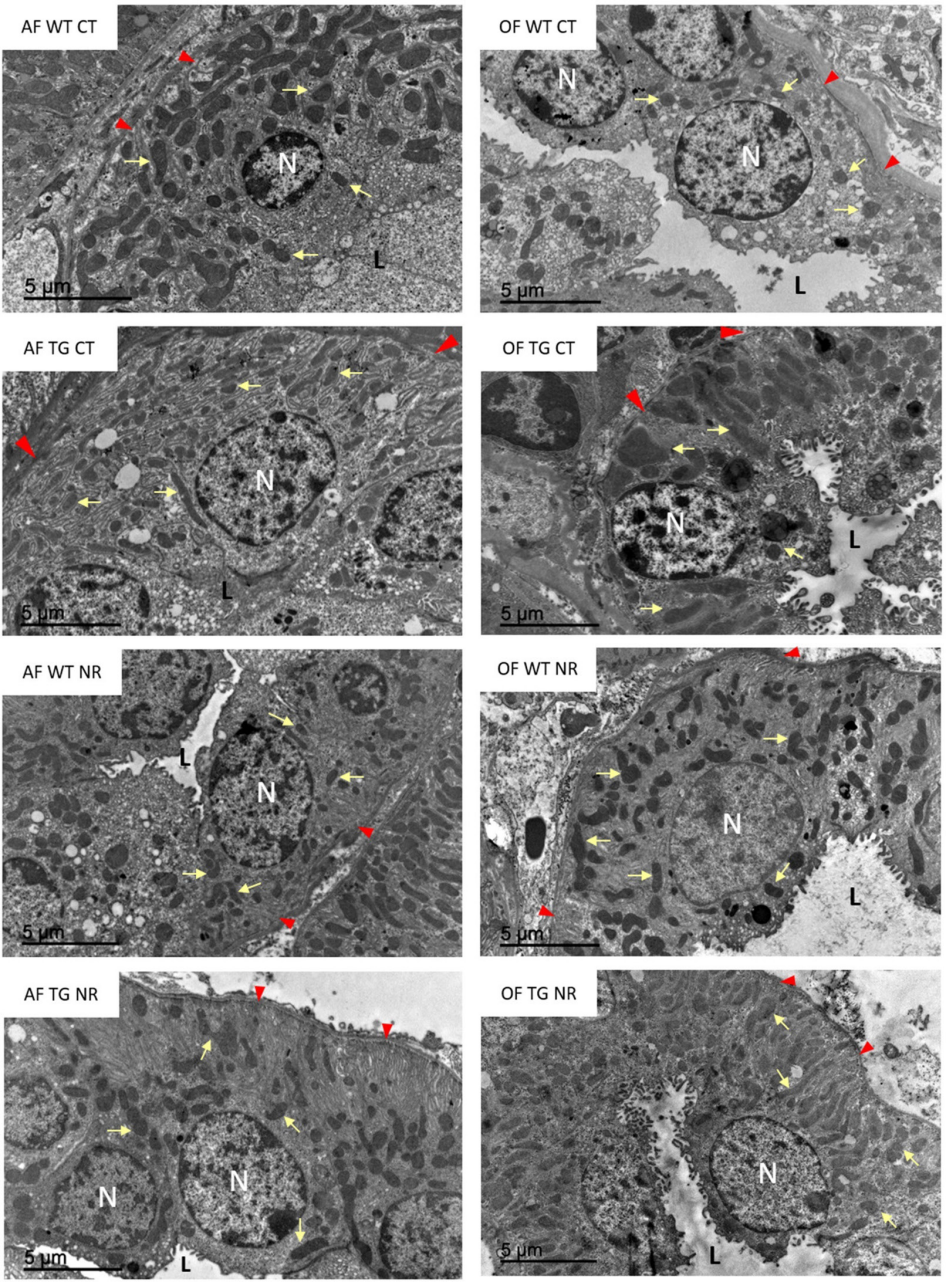
The examples of distal convoluted tubule epithelial cells from female mice from the different experimental groups. The panels on the left display images of adult mice, whereas those on the right correspond to old animals. Each panel includes a tag indicating the experimental group. Some examples of mitochondria are marked with yellow arrows while red arrow heads indicate basal folds of the plasma membrane. L, tubular lumen; N, nucleus.

At the electron microscope, distal tubules from our in vivo model showed a typical morphology consisting of a simple cuboidal epithelium surrounding a lumen practically free of microvilli. The cells displayed central or apical nuclei with extensive folds of the plasma membrane at the basal domain, and numerous mitochondria mainly located at the same pole. Figures 3 and 4 show examples of tubular cells from the different experimental groups illustrating the type of material used in this study.

### 7.1 Mitochondrial area

**FIGURE 5:**
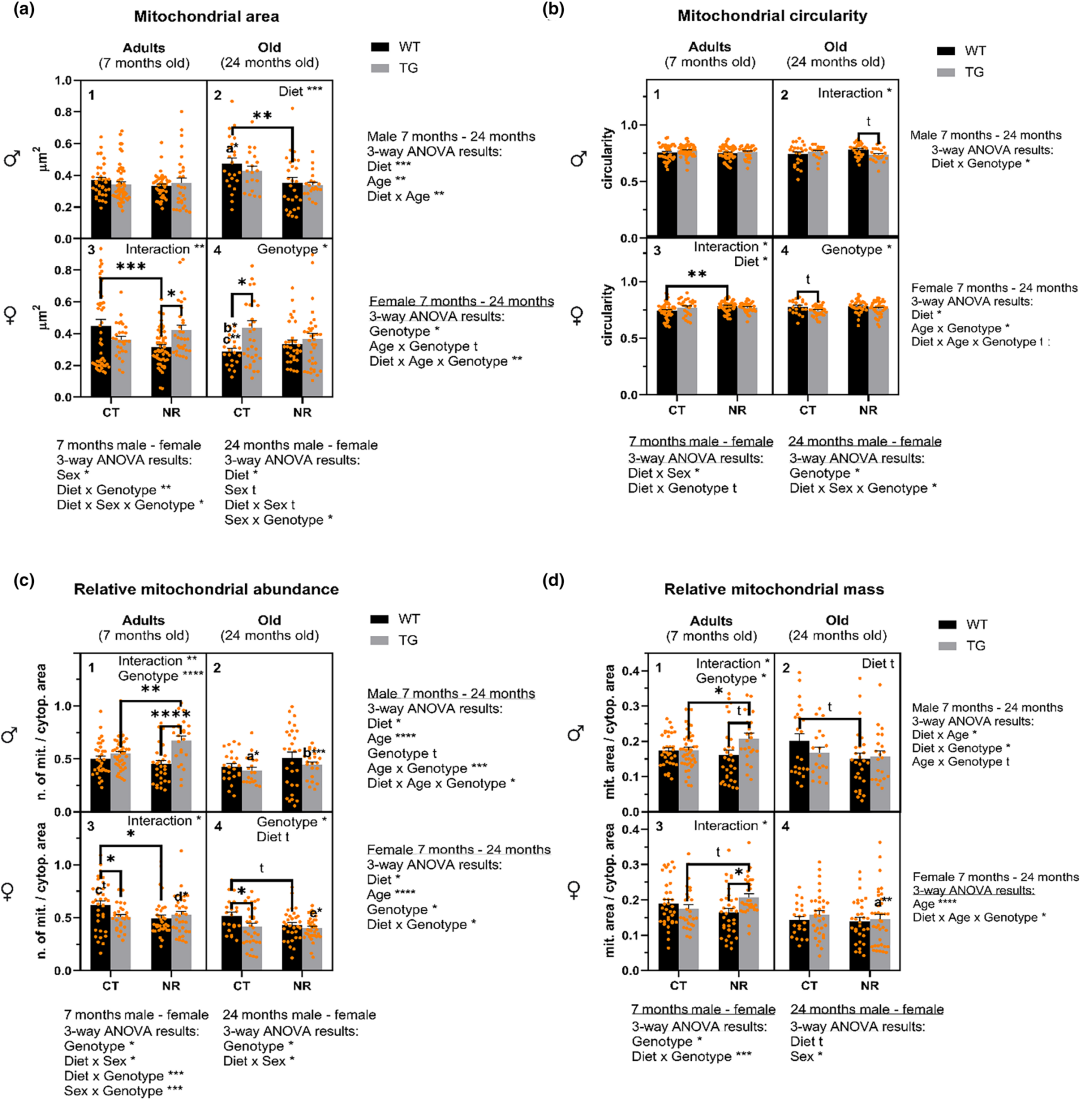
Mitochondrial area (a) and circularity (b), relative mitochondrial abundance (c) and mass (d) in distal convoluted tubule cells of male and female mice subjected to different experimental conditions. Mitochondrial area is expressed in μm^2^. Mitochondrial circularity is expressed with values from 0 to 1 where a value of 1 indicates a perfect circle. Abundance data are expressed as the number of mitochondria per cytoplasm unit area. Mass data are expressed as the sum of mitochondrial area/cytoplasmic area (see Materials and Methods). In (a–d), the upper panels (1 and 2) display the results of the evaluated parameters in males, while the lower panels (3 and 4) show those of females. Values from adult (7‐months‐old) animals are presented in the left panels (1 and 3), whereas those from old (24‐months‐old) mice are depicted in the right panels (2 and 4). The black bars correspond to WT mice, while the gray ones represent TG animals overexpressing CYB5R3. CT denotes mice fed the control diet, and NR indicates mice fed the NR‐diet. Orange dots represent individual values. The results of statistical analyses comparing different ages within each sex are displayed on the right side of panels 2 and 4. Likewise, the results of statistical analyses comparing animals of different sexes with the same age are presented beneath panels 3 and 4. The results of the 2‐way ANOVA for each subgroup are included within each panel. Connected bars and letters above the bars indicate the results of Sidak‐type post hoc tests. t, tendency (*p* < 0.1); *p < 0.05; **p < 0.01; ***p < 0.001; ****p < 0.0001. In (a), a*p < 0.05 versus AM WT CT; b*p < 0.05 versus AF WT CT; c**p < 0.01 versus OM WT CT. In (c), a*, *p* < 0.05 versus AM TG CT; b***, *p* < 0.001 versus AM TG NR; c^t^, *p* < 0.1 versus AM WT CT; d*, *p* < 0.05 versus AM TG NR; and e*, *p* < 0.05 versus AF TG NR. In (d), a**, *p* < 0.01 versus AF TG NR.

As shown in Figure 5a, age, sex, genotype, and diet caused changes in mitochondrial size in DCT cells from our in vivo model. In AM, mitochondrial area was similar in all the experimental groups (Figure 5a1) and aging increased mitochondrial size, especially in WT animals that had been fed the CT diet (Figure 5a1,2, *p* < 0.05). Conversely, NR dietary supplementation decreased average mitochondrial area regardless of age and genotype (Figure 5a1,2, *p* < 0.001). This change was driven by the reduced mitochondrial size in old mice due to NR supplementation (Figure 5a2, *p* < 0.001), with a more pronounced effect in those of WT genotype (Figure 5a2, *p* < 0.01).

For females, three‐way ANOVA showed interactions among variables (Figure 5a3,4, *p* < 0.01), particularly age x genotype (Figure 5a3,4, *p* < 0.1). In AF, CYB5R3 overexpression induced no significant change in mice fed the CT diet, whereas a significant increase was detected in those mice on a NR diet (Figure 5a3, *p* < 0.01), indicating a diet x genotype interaction (Figure 5a3, *p* < 0.01). NR supplementation reduced mitochondrial size in WT AF (Figure 5a3, *p* < 0.001). Aging also reduced mitochondrial area in WT females on a CT diet (Figure 5a3,4, *p* < 0.05). Overexpression of CYB5R3 increased mitochondrial size in OF (Figure 5a4, *p* < 0.05), especially in those on a CT diet (see Figure 5a4, *p* < 0.05).

An interaction between diet, sex and genotype was detected in adults, both for males and females, (Figure 5a1,3, *p* < 0.05), being more evident the diet x genotype interaction (Figure 5a1,3, *p* < 0.01). Furthermore, females exhibited a larger mitochondrial size than males (Figure 5a1,3, *p* < 0.05). OM tended to have larger mitochondria than OF (Figure 5a2,4, *p* < 0.1), particularly in the case of WT CT mice (Figure 5a2,4, *p* < 0.01), and NR supplementation also induced significant changes (Figure 5a2,4, *p* < 0.05). A diet x sex interaction was observed in old mice, since a decrease of mitochondrial size due to NR was observed in males but not in females (Figure 5a2,4, *p* < 0.1). Finally, a sex x genotype interaction was also detected. Here, mitochondrial size was increased by CYB5R3 overexpression in OF but decreased in OM (Figure 5a2,4).

### 7.2 Mitochondrial circularity

Regarding mitochondrial circularity, there was a diet x genotype interaction in males across all ages (Figure 5b1,2, *p* < 0.05) that was especially evident in old animals (Figure 5b2, *p* < 0.05). Moreover, we evidenced in females an increase in circularity related with NR diet regardless of age and genotype (Figure 5b3,4, *p* < 0.05). This effect was more conspicuous in AF (Figure 5b3, *p* < 0.05) and especially in those of WT genotype (Figure 5b3, *p* < 0.01), showing thus an age x genotype interaction (Figure 5b3,4, *p* < 0.05). A tendency towards a trivariate interaction among diet, age, and genotype was also found (Figure 5b3,4, *p* < 0.1). CYB5R3 overexpression reduced circularity in OF (Figure 5b4, *p* < 0.05).

When changes of circularity were examined among sexes, a diet x sex interaction emerged in adult animals (Figure 5b1,3, *p* < 0.05). NR diet increased circularity in AF (Figure 5b3, *p* < 0.1), while decreasing it in AM (Figure 5b1). With respect to sex‐independent changes, a diet x genotype interaction was noticeable in adult animals (Figure 5b1,3, *p* < 0.1), especially in females (Figure 5b3, *p* < 0.05). CYB5R3 overexpression reduced circularity in old animals (Figure 5b2,4, *p* < 0.05), being this effect particularly evident in OF (Figure 5b4, *p* < 0.05). Among OM, this effect was only remarkable with NR supplementation (Figure 5b2, *p* < 0.1). Finally, an interaction among diet, sex, and genotype was observed in older animals (Figure 5b2,4, *p* < 0.05).

### 7.3 Mitochondrial relative abundance (Na)

Mitochondrial mass and relative abundance also changed across the different variables (Figure 5c,d). In males, the relative mitochondrial abundance (Na) was influenced by NR supplementation. Thus, Na was higher in mice fed the NR diet compared to those fed the CT diet (Figure 5c1,2, *p* < 0.05). On the other hand, an age x genotype interaction was observed (Figure 5c1,2, *p* < 0.001). CYB5R3 overexpression led to a significant increase of Na in adult mice (Figure 5c1, *p* < 0.0001), while the opposite effect was found in old animals, which caused old TG mice to be significantly different from TG adults (Figure 5c1,2, *p* < 0.0001). Moreover, AM exhibited divergent response patterns to CYB5R3 overexpression depending on whether the diet had been supplemented or not with NR (Figure 5c1, *p* < 0.01). Thus, the effect of transgenesis was more pronounced in those mice fed the NR diet (Figure 5c1, *p* < 0.0001), causing relative mitochondrial abundance to be higher in AM TG NR compared to AM TG CT group (Figure 5c1, *p* < 0.01). Altogether, aging led to decreased Na (Figure 5c1,2, *p* < 0.0001), and a triple interaction diet x age x genotype was observed in males (Figure 5c1,2, *p* < 0.05).

In females, aging also caused a decrease in Na (Figure 5c3,4, *p* < 0.0001) and NR supplementation further decreased relative mitochondrial abundance regardless of age and genotype (Figure 5c3,4, *p* < 0.05), as well as genotype regardless of age and diet (Figure 5c3,4, *p* < 0.05). This main effect of NR diet was observed in OF as well, being this effect mainly due to the changes observed in WT mice (Figure 5c4, *p* < 0.1). The main effect of transgenesis was also observed in old mice (Figure 5c4, *p* < 0.05). Moreover, in both AF and OF, CYB5R3 overexpression effect was more prominent in animals fed the CT diet (Figure 5c3,4, *p* < 0.05). Despite this, the results also indicated an interaction diet x genotype (Figure 5c3,4, *p* < 0.05), an effect that was also observed in AF, revealing a complex relationship between these variables (Figure 5c3, *p* < 0.05). Here, in AF WT, NR diet produced the same decreasing effect on Na observed in old animals of the same genotype (Figure 5c3,4, *p* < 0.05 and p < 0.1, respectively). Conversely, AF TG NR group exhibited higher Na than AF TG CT or AF WT NR groups, which explained the previously reported interaction, and caused a difference between AF TG and OF TG on an NR diet, with the latter showing lower Na values (Figure 5c3,4, *p* < 0.05).

Comparing AM and AF, a diet x sex (Figure 5c1,3, *p* < 0.05) and a sex x genotype interaction (Figure 5c1,3, *p* < 0.001) was observed. Both CYB5R3 overexpression and NR supplementation enhanced Na in AM, while the opposite was observed in AF. Thus, AM TG NR mice exhibited higher Na than their female counterparts (Figure 5c1,3, *p* < 0.05). In terms of aging, the same interaction between diet and sex that had been observed in adult animals was also detected in aged mice (Figure 5c2,4, *p* < 0.05). On the other hand, transgenesis induced consistent changes regardless of the sex and diet, resulting in decreased Na (Figure 5c2,4, *p* < 0.05).

### 7.4 Mitochondrial relative mass (a/a)

Relative mitochondrial mass (a/A) results in AM and OM showed complex interactions as diet x genotype (Figure 5d1,2, *p* < 0.05), age x genotype (Figure 5d1,2, *p* < 0.1), and diet x age (Figure 5d1,2, *p* < 0.05). This parameter was higher in AM TG than in AM WT independently of diet (Figure 5d1, *p* < 0.05), which was especially pronounced in those mice on the NR‐diet, which led to a diet x genotype interaction (Figure 5d1, *p* < 0.05). Furthermore, AM TG NR showed higher a/A than AM TG CT (Figure 5d1, *p* < 0.05) and AM WT NR (Figure 5d1, *p* < 0.1) groups. Additionally, NR supplementation reduced a/A in aged mice, especially in those of WT genotype (Figure 5d2, *p* < 0.1), but the reverse was found in adult animals (diet × age interaction). In females, this parameter changed with age as OF exhibited lower a/A than AF (Figure 5d3,4, *p* < 0.0001), particularly in the case of OF TG NR mice (Figure 5d3,4, *p* < 0.01). An interaction between diet, age and genotype was also observed, showing a complex influence of the three variables (Figure 5d3,4, *p* < 0.05), especially in AF, which showed a specific interaction between diet and genotype (Figure 5d3, *p* < 0.05). While transgenesis induced a decrease of a/A in AF fed the CT diet, it induced the opposite effect in AF fed the NR‐diet. Thus, AF TG NR showed a higher relative mitochondrial mass than AF TG CT (Figure 5d3, *p* < 0.1) and AF WT NR groups (Figure 5d3, *p* < 0.05).

When analyzing the different groups of adult mice, we observed genotype triggered changes regardless of sex (Figure 5d1,3, *p* < 0.05), being these differences largely guided by the effect of transgenesis in AM and in AF TG fed the NR‐ diet. A diet x genotype interaction was also observed (Figure 5d1,d3, *p* < 0.001), as previously described in both sexes. NR supplementation tended to decrease a/A in OM and OF, especially in the former (Figure 5d2,4, *p* < 0.1). Furthermore, increased a/A values were found in OM when compared with OF (Figure 5d2,4, *p* < 0.05).

### 7.5 Mitochondria‐endoplasmic reticulum contact sites (MERCS)

**FIGURE 6:**
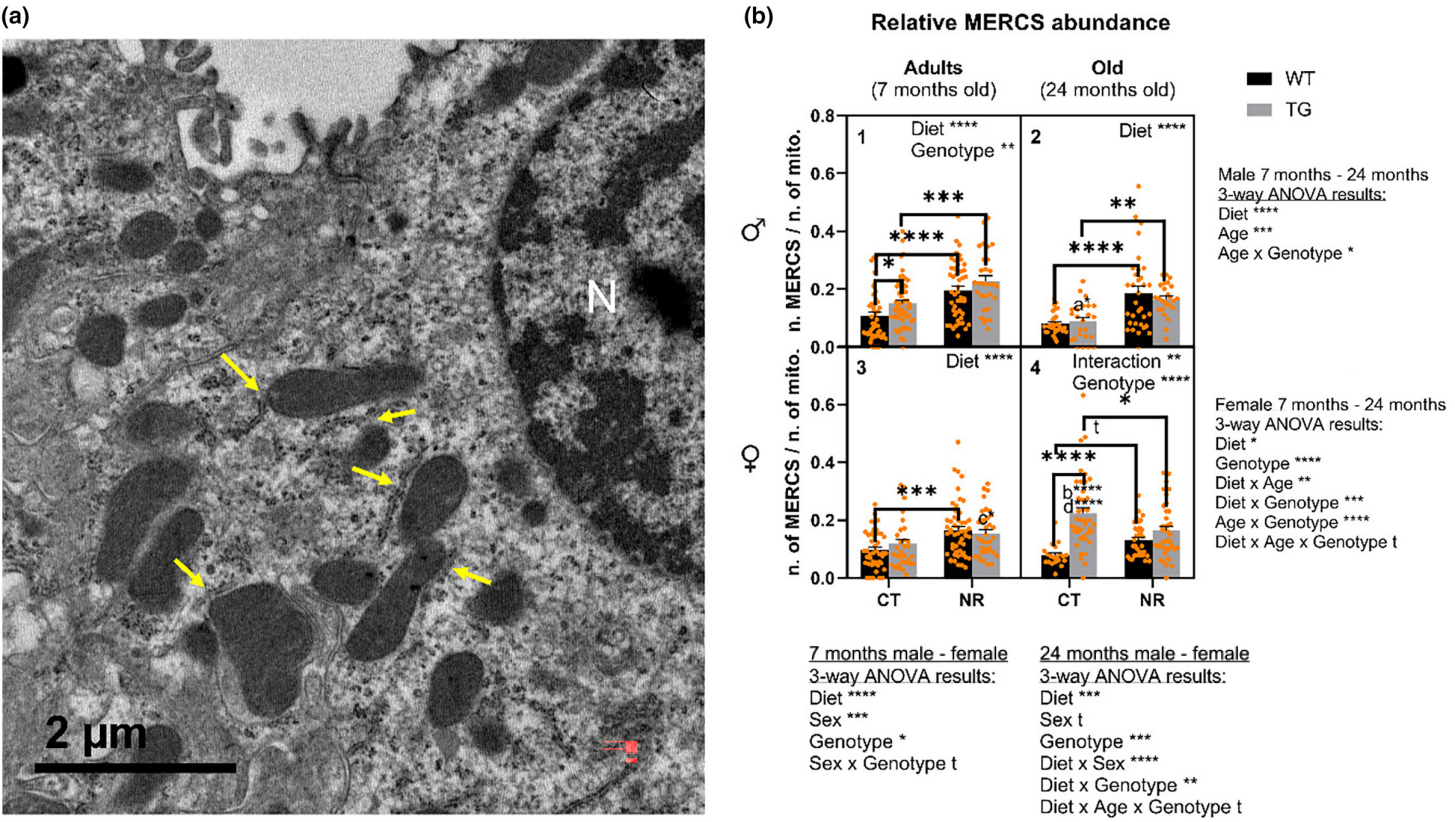
Mitochondria‐endoplasmic reticulum contact sites (MERCS) abundance in distal convoluted tubule cells under the different experimental conditions. (a) Shows some examples of MERCS marked with yellow arrows. (b) Shows relative MERCS abundance. MERCS abundance data are expressed as the number of MERCS per mitochondria (see Materials and Methods). See Figure 5 caption for a description of the panel arrangement. In (b); t, tendency (*p* < 0.1); *p < 0.05; **p < 0.01; ***p < 0.001; ****p < 0.0001; a*, *p* < 0.05 versus AM TG CT; b****, *p* < 0.0001 versus AF TG CT; c*, *p* < 0.05 versus AM TG NR; d****, *p* < 0.0001 versus OM TG CT.

In the electron microscopy images, MERCS appeared as a juxtaposition of a mitochondria with endoplasmic reticulum cisternae varying in distance and length (see Figure 6a for some MERCS examples). In our samples, the distance and length of scored MERCS depended on sex, age, genotype and diet (see Figure S2). In males, the MERCS distance was increased due to aging (Figure S2a1,2, *p* < 0.05). In contrast, NR supplementation and CYB5R3 overexpression decreased the mitochondria‐endoplasmic reticulum distance (Figure Sa1,2, *p* < 0.0001 and p < 0.001, respectively). In females, this parameter decreased with age (Figure S2a3,4, *p* < 0.1). Here, transgenesis increased MERCS distance in OF compared to their wild‐type counterparts (Figure S2a4, *p* < 0.01). On the other hand, aging decreased MERCS length in male and female animals on a CT diet (Figure S2b2,4, *p* < 0.01), while this parameter remained unaltered in animals under a NR‐supplemented diet (Figure S2b2,4).

As displayed in Figure 6b1,2, NR supplementation induced an increase of MERCS abundance in males (Figure 6b1,2, *p* < 0.0001), while the effect of CYB5R3 overexpression was dependent on age (Figure 6b1,2, *p* < 0.05). On the other hand, a reduction of MERCS abundance was observed with aging in males (Figure 6b1,2, *p* < 0.001), being this effect particularly pronounced in the OM TG CT group (Figure 6b2, *p* < 0.05). Both NR‐diet and CYB5R3 overexpression increased MERCS number in AM (Figure 6b1, *p* < 0.0001 and p < 0.01, respectively). Changes induced by transgenesis were more evident in mice fed the CT diet (Figure 6b1, *p* < 0.05), while the effect of NR supplementation was clearly observed in both genotypes (Figure 6b1, *p* < 0.0001 and *p* < 0.001 for WT and TG mice, respectively). NR‐diet induced significant alterations in OM (Figure 6b2, *p* < 0.0001). Again, modifications triggered by NR‐diet were observed in both WT and TG mice (Figure 6b2, *p* < 0.0001 and *p* < 0.001, respectively). In females, we observed a complex interaction between age, genotype, and diet (see Figure 6b3,4‐way ANOVA results). However, a main effect of diet (Figure 6b3,4, *p* < 0.05) and genotype (Figure 6b3,4, *p* < 0.0001) arose, showing and increase of MERCS abundance. NR‐diet increased MERCS counts in AF (Figure 6b3, *p* < 0.0001), especially in those of WT genotype (Figure 6b3, *p* < 0.001). In OF, transgenesis significantly increased MERCS (Figure 6b4, *p* < 0.0001). However, a genotype x diet interaction was also observed (Figure 6b4, *p* < 0.01). Thus, transgenesis effect in OF was more intense in those fed the CT diet (Figure 6b4, *p* < 0.0001).

Differences between males and females were also conveyed in both adult (Figure 6b1,3, *p* < 0.0001) and old (Figure 6b2,4, *p* < 0.1) mice. Here, we evidenced main effects of diet and genotype independent of sex, as well as the interaction of theses variables with sex, further accentuating the differences between sexes described above (see 3‐way ANOVA results for comparison between adult and old males and females).

## 8 DISCUSSION

The kidney undergoes changes during aging. However, this organ exhibits marked physiological and structural dimorphism between sexes (Tahaei et al., 2020), and these changes affect males and females differently. However, as far as we know, there is a lack of information regarding changes in the mitochondrial morphology, abundance and mass, as well as in MERCS, of DCT cell related to aging, sex, or in response to anti‐aging interventions. We have thoroughly characterized these processes in the present study and obtained relevant biological insights.

The analyzed transcriptomic data revealed remarkable differences between both sexes at DCT cells. As previously reported, key markers of these cells showed an opposite pattern of gene expression levels associated with homeostatic and transport processes, principally involving sodium, calcium, potassium, and chloride (McCormick & Ellison, 2015). On the other hand, transcripts of the anti‐aging protein Klotho (Kl) decreased with age, in line with previous studies (Buchanan et al., 2020). However, a more acute situation was observed in females. This divergent effect of aging between sexes was also detected among the other analyzed markers when differences between adult and old males and females were evaluated. These items highlight a different background for each described experimental conditions, which may offer perspective on the observed differences with anti‐aging interventions on these cells. Furthermore, some of these markers that exhibited differential expression have been also associated with ATP‐dependent processes (Alexander et al., 2015; Hamilton & Devor, 2012), further supporting the diverse response of mitochondrial populations from DCT cells to the imposed conditions.

Under our experimental conditions, mitochondria were smaller in AM compared to AF, which could represent a novel element of sexual dimorphism (Delaney et al., 2018; Tahaei et al., 2020). Furthermore, while mitochondrial size remained relatively constant in males across the different experimental groups, CYB5R3 overexpression and NR supplementation decreased mitochondrial size in AF. However, these changes were reversed when the interventions were combined. As previously reported, CYB5R3 overexpression also reduced mitochondrial size in skeletal muscle white fibers (López‐Bellón et al., 2022). Nevertheless, a separate study involving males and females showed that mitochondrial size decreased in males but significantly increased in females, further accentuating a sex‐dependent response to this intervention (Sánchez‐Mendoza et al., 2023).

The effect of aging on mitochondrial size varies depending on the cell/tissue type. Thus, in previous studies focused on male mice, an increase in mitochondrial volume in hepatocytes (Khraiwesh et al., 2014) and in PCT cells (Calvo‐Rubio et al., 2016) was reported, while a decrease was observed in subsarcolemmal mitochondria from type I skeletal muscle fibers (Gutiérrez‐Casado et al., 2019). Interestingly, in these cases, the type of fat included in the diet significantly affected determining changes of mitochondrial size with aging, indicating the adaptability of this organelle to the conditions imposed by diet. Additionally, in other study conducted on skeletal muscle from males, it was found that aging decreased the size of mitochondria in type I and II fibers (López‐Bellón et al., 2022). In DCT cells, we found that the effect of aging on mitochondrial size in males followed an increasing pattern like that described by Khraiwesh et al. (2014) in hepatocytes and by Calvo‐Rubio et al. (2016) in PCT cells. However, the opposite effect was detected in females, which followed a pattern of changes like that described in skeletal muscle from males (Gutiérrez‐Casado et al., 2019; López‐Bellón et al., 2022).

In addition to the above‐mentioned issues, it is necessary to consider the distinctions between tissues and cell types according to their unique functions. In this context, a tissue, substrate, and site‐specific pattern of mitochondrial ROS generation has been described in muscle, liver, and kidney (Tahara et al., 2009). Also, the mitochondria of PCT have been reported to be in a higher state of oxidation and to produce more ROS than those of DCT (Hall et al., 2009). These characteristics may contribute to explaining the conditions of the tissues and nephron segments that have been compared.

Cells in the DCT contain abundant mitochondria (McCormick & Ellison, 2015; Subramanya & Ellison, 2014) which can be accounted by the high number of active transporters and the need for fuel in the form of ATP. Moreover, the levels, function and capacity of these transporters are regulated by the availability of the ions to be transported. As a result, the availability of these ions and the control of their transport can ultimately impact mitochondrial morphology and population (Subramanya & Ellison, 2014). Furthermore, the regulation of ion transport in DCT is influenced by sex hormones. So, male mice have been shown to have higher urinary calcium excretion than females, which is associated with lower expression of different calcium transporters, a situation also found in the transcriptomic data displayed in our study. This phenomenon has been linked to testosterone levels in the males. Interestingly, orchiectomy increased the abundance of these transporters. On the contrary, estrogens have a stimulatory effect on the expression of Ca^2+^ transport proteins. Thus, postmenopausal women with estrogen deficiency have been associated with a negative calcium balance and an increased risk of osteoporosis (McCormick & Ellison, 2015). Considering all these items, it is plausible to suggest that hormonal dynamics could help to explain the observed results in DCT, with aging leading to increased mitochondrial area in males while causing a reduction in females.

As we show here, dietary and genetic interventions influenced mitochondrial size in both sexes. The combination of CYB5R3 overexpression and NR‐dietary supplementation largely blunted the changes induced by aging at mitochondrial size level and even induced a mild synergistic effect in males. However, once again, disparities between sexes were apparent. In females, these alterations primarily stemmed from CYB5R3 overexpression, increasing mitochondrial size. In contrast, in males, this effect was primarily a consequence of NR dietary intervention, leading to a reduction in mitochondrial size. In this sense, attenuation of aging‐related changes by these anti‐aging interventions has been previously reported. As mentioned above, aging induced a reduction in mitochondrial size in muscular white and red fibers, and CYB5R3 overexpression increased mitochondrial size in old mice (López‐Bellón et al., 2022). On the other hand, saving distances from our experimental conditions, NR supplementation increased mitochondrial cristae content in male mice fed a high‐fat diet, while mitochondrial size remained unaltered in brown adipose tissue (Cantó et al., 2012). These and our results indicate a tissue‐dependent adaptive response of mitochondrial size to the conditions imposed by age, sex, genotype, and diet.

Differentially expressed CYB5R3‐, and NAD^+^ biosynthesis‐related genes also revealed a distinct sexual dimorphism, as observed in the transcriptomic data results. Levels of Cyb5r3 and its associated genes were lower in females. On the contrary, males exhibited higher expression levels of NAD^+^ biosynthesis genes, with particular emphasis on Nmrk1. The protein encoded by Nmrk1 initiates the conversion of NR to NAD^+^ (Ratajczak et al., 2016). Remarkably, this pattern was consistent with the effects triggered by both CYB5R3 overexpression and NR supplementation in males and females. Thus, it is reasonable that the most prominent effects of CYB5R3 overexpression were observed in female animals, which exhibited lower levels of Cyb5r3, while the most noticeable effects of NR supplementation were seen in males, which displayed higher levels of NAD^+^ biosynthesis genes.

Far from being static structures, mitochondria continuously change shape due to fission and fusion processes. Once again, our results evidenced the influence of age, sex, diet, and genotype in the observed mitochondrial circularity in DCT. Thus, it can be assumed that these alterations in mitochondrial morphology could be attributable to changes in mitochondria dynamics. In this regard, it is interesting to note that in hepatocytes and PCT cells, aging exerts differential changes in mitochondrial circularity, with an increase in the former (Khraiwesh et al., 2014) and a decrease in the latter (Calvo‐Rubio et al., 2016), highlighting again the existence of cell/tissue specificity.

Aging and anti‐aging interventions induce changes in the size and shape of mitochondria and in mitochondrial content following tissue‐specific patterns. For example, aging led to a decrease in mitochondrial mass in hepatocytes and in PCT cells from male mice (Khraiwesh et al., 2014 and Calvo‐Rubio et al., 2016, respectively). These results contrast with those reported by Gutiérrez‐Casado et al. (2019) in type I fibers of skeletal muscle, where intermyofibrillar mitochondria increased their mass in aged males. In a recent study focused on male mice subjected to a ketogenic diet and using various approaches to determine mitochondrial mass, Zhou et al. (2021) reported differential changes depending on the analyzed organ or tissue, confirming the tissue‐specific nature of mitochondrial mass adaptations in response to different interventions.

It has been established that the mitochondrial population can undergo notable changes in response to various liver and skeletal muscle interventions. Thus, NR supplementation increased the relative mitochondrial number and area in the muscles of healthy individuals (Lapatto et al., 2023). Also, in male mice on a high‐fat diet, NR increased mitochondrial DNA copies in muscles (Cantó et al., 2012). Nicotinamide mononucleotide increased mitochondrial DNA copies in the muscles of female mice fed a high‐fat diet, while reducing them in liver (Uddin et al., 2017). Interestingly, in skeletal muscle of male mice, CYB5R3 overexpression has been shown to reverse the modifications associated with aging in both relative mitochondrial abundance and mass of white fibers, as well as the mitochondrial mass of red fibers (López‐Bellón et al., 2022). In accordance with these results, in our study NR supplementation and CYB5R3 overexpression tended to alleviate the effects of aging on parameters related to mitochondrial morphology and mass.

Concerning mitochondrial mass and abundance, our findings in DCT demonstrated a decline with aging, as well as with transgenesis and NR supplementation in AF. The combination of both interventions promoted an increase in mitochondrial parameters in adults of both sexes, while the diet increased mitochondrial abundance in OM and induced the opposite effect in OF. As suggested previously, these differences between males and females, as well as the divergent response to the anti‐aging interventions analyzed here, can probably be linked to sex hormones and their level variations with aging, and to the differences observed in key DCT, CYB5R3, and NAD^+^ biosynthesis‐related markers between sexes and ages in mice.

MERCS have been also recognized as vulnerable to aging‐driven changes. As organisms age, the number of MERCS and the expression levels of genes associated with these contact sites decrease (Vue et al., 2023). Our study also revealed a decline in MERCS features in DCT cells with aging. Remarkably, both CYB5R3 overexpression and NR supplementation enhanced MERCS abundance in adult and old mice of both sexes, and palliated age‐associated alterations in MERCS distance and length. These changes might be associated with improved redox signaling regulation, mitochondrial DNA replication, dynamics, and mitophagy (Moltedo et al., 2019). So, our interventions may impact crucial processes for maintaining mitochondrial population health, through optimizing function, dynamics and ROS production (Willems et al., 2015), also reducing mitochondrial DNA mutation accumulation (Sanchez‐Contreras et al., 2023) or even promoting the removal of aberrant mitochondria through autophagy (Ryter et al., 2013).

Taken together, the results described in this study have indicated the existence of different mechanisms of adaptation of mitochondrial morphology and mass, as well as MERCS features, depending on age, sex, CYB5R3 overexpression and NR dietary supplementation in DCT cells. Overall, combining CYB5R3 overexpression and NR dietary supplementation effectively palliates aging‐related changes. Notably, in males, the effects can be mainly attributed to NR supplementation, whereas in females, they are predominantly linked to transgenesis – being these results probably due to higher expression levels of NAD^+^ biosynthesis genes in males and the lower levels of CYB5R3‐related genes in females, and also to the differences between key DCT markers. Here, it is crucial to emphasize that the impact of these interventions on age‐related changes is notably complex, involving intricate interactions between molecular changes, cellular physiology adaptations, and exhibited phenotypes, thus complicating the full understanding of the triggered processes (Skowronska‐Krawczyk, 2023). Nevertheless, this stresses the importance of employing sex and tissue‐specific approaches to thoroughly tackle the diverse aspects of aging.

## AUTHOR CONTRIBUTIONS

Rd.C., J.A.G.R., and J.M.V conceived and designed the project. Rd.C. developed the transgenic line of CYB5R3‐overexpressing mice. L.M.S.M. and M.P.R. were responsible for raising, maintaining, and genotyping the mouse colony. M.P.R. performed transcriptomic analysis. M.P.R., L.M.S.M., A.G.V., A.M.M., N.B., and M.I.B. carried out sample processing, data collection, and electron microscopy analysis. C.P.S. and J.A.M. provided valuable advice. J.A.M., Rd.C., and J.M.V. provided the resources and funding. M.P.R. and J.A.G.R. interpreted the results and wrote the original manuscript draft, incorporating feedback from all contributors.

## FUNDING INFORMATION

Funding for open access charge: Universidad de Córdoba/Consorcio de Bibliotecas Universitarias de Andalucía (CBUA).

## CONFLICT OF INTEREST STATEMENT

The authors declare no conflicts of interest.

## Supporting information


Data S1.

## Data Availability

The data supporting our study's findings is available from the corresponding author upon reasonable request. The transcriptomic data that support the findings of this work are openly available at GEO database (reference number GSE175854).
