# Supplementary material for: Cytochrome b 5 reductase 3 overexpression and dietary nicotinamide riboside supplementation promote distinctive mitochondrial alterations in distal convoluted tubules of mouse kidneys during aging
Source: Aging Cell. 2024 Jul 12;23(11):e14273. doi: 10.1111/acel.14273 (PMC11561664; doi:10.1111/acel.14273)
Supplement: Supplementary file 1 — Data S1. [file ACEL-23-e14273-s001.docx]

**Supplementary information**

**transmission electron microscopy SAMPLES preparation PROTOCOL**

The kidneys were extracted and quickly washed in 0.1 M sodium cacodylate buffer at pH 7.2. Then, samples from renal cortex (about 4-5 pieces of 1 mm^3^) were fixed in 2.5 % glutaraldehyde in 0.1 M sodium cacodylate buffer pH 7.2 for 1 h at room temperature and 24 h at 4 °C, washed twice in cacodylate buffer (15 min each) and post fixed in 1 % osmium tetroxide for 1 h at 4 °C in the same buffer. The pieces were then dehydrated in an ascendent ethanol series (50°, 70°, 90° and 100° x 3 times in steps of 20 min each), transferred to propylene oxide (two passes of 20 min each) and sequentially infiltrated in EMbed 812 resin (EMS; Hatfiel, PA; USA). We used the sequence propylene oxide:resin 2:1, 1:1, and 1:2 throughout 24 h (8 h each). The resin was prepared following the recommendation of the manufacturer for “medium hardness”. Afterwards, samples were transferred to pure resin for 24 h. Then, blocks were formed in silicon molds with fresh resin for 48 h at 65 °C. After trimming the blocks, semi-thick sections (0.5 μm thick) and ultra-thin sections (~60 nm thick) were obtained for studies with light and electron microscope, respectively. Both types of sections were obtained using a Reichert-Jung ultramicrotome (Germany) and a diamond knife (Diatome, Switzerland). Semi-thick sections were mounted on glass slides, and then stained with 1% toluidine blue in 1% borax solution for one minute at 65°C for an initial evaluation to select the blocks that would be sectioned at the ultramicrotome. Ultra-thin sections were mounted on nickel grids and contrasted for 2 minutes with 2% uranyl acetate, washed in distilled water, and with "ready-to-use" lead citrate (EMS, USA) for 2 minutes.

**Supplementary list 1.- Key DCT-related genes.** The list includes 82 unique genes identified from the top 50 meta-markers of DCT cells and the top 50 meta-markers of DCT-like DCT-CNT cells according to Novella-Rausell *et al.* (2023).

Slc15A3

Wnk1

Tmem55B

Klhl3

Abca13

Slc16a7

Sgms2

Pvalb

Sfrp1

Trpm6

Wnk4

Ptgfr

Lhx1

Cwh43

Tox3

Uroc1

Emx1

Hoxb5os

Kctd1

Fam81a

Defb1

Ppp1r1a

Tmem213

Pgam2

Cadps2

Clu

Tmem72

Clcnkb

Hoxd8

Cables1

Cnnm2

Kcnj10

Rab27a

Aebp1

Syt17

Fads6

Nup93

Ank2

Kng2

Trpm7

Mt1

Kcnj1

Wfdc15b

Calb1

Ckb

Ier3

Mt2

Lhx1os

Pcolce

Mfsd5B-1

Slc8a1

Kl

Klk1

Phactr1

S100g

Dach1

Tdrd3

Tsc25D1

Acss3

Cdk14

Tsc25D2

Papss1

AI838599

Ltc4s

Slc5A9

Atp5B4

Flvcr1

4933406I18Rik

Sorbs2

Gm26542

9330159M07Rik

Ryr2

Pde10a

Dsg2

Slc5B-13

E230016K23Rik

Cntfr

Stk35A

Efhc2

Sgsm1

Mecom

Vdr

**Supplementary list 2.- Key CYB5R3-related genes.** The list includes genes from CYB5R3 and its 5 most confident interactors according to the STRING database.

Cyb5r3

Cmah

Cyb5a

Cyb5b

Dhodh

Suox

**Supplementary list 3.- Key NAD^+^ biosynthesis-related genes.** The list includes 12 genes involved in the NAD^+^ synthesis pathway according to Xie *et al.* (2020).

Ido1

Ido2

Tdo2

Qprt

Naprt

Nadsyn1

Nmrk1

Nmrk2

Nmnat1

Nmnat2

Nmnat3

Nampt

**Supplementary Table 1.- Number of animals, CDT cells, mitochondria, and MERCS scored in this study for the different experimental groups.** AM: adult males; AF: adult females; OM: old males; OF: old females. WT: wild type; TG: transgenic; CT: control diet; NR: nicotinamide riboside supplemented diet.

| **Experimental Group** | **Number of animals** | **Number of DCT cells** | **Number of scored mitochondria** | **Number of scored MERCS** |
| --- | --- | --- | --- | --- |
| **AM WT CT** | 7 | 39 | 1,900 | 260 |
| **AM TG CT** | 5 | 56 | 2,098 | 316 |
| **AM WT NR** | 5 | 46 | 1,445 | 316 |
| **AM TG NR** | 4 | 30 | 1,535 | 345 |
| **OM WT CT** | 4 | 23 | 648 | 76 |
| **OM TG CT** | 4 | 19 | 617 | 56 |
| **OM WT NR** | 5 | 28 | 1,050 | 160 |
| **OM TG NR** | 4 | 21 | 662 | 205 |
| **AF WT CT** | 6 | 48 | 1,590 | 185 |
| **AF TG CT** | 5 | 28 | 1,299 | 224 |
| **AF WT NR** | 5 | 54 | 1,581 | 273 |
| **AF TG NR** | 5 | 32 | 1,276 | 233 |
| **OF WT CT** | 4 | 21 | 557 | 65 |
| **OF TG CT** | 5 | 32 | 806 | 281 |
| **OF WT NR** | 4 | 31 | 821 | 128 |
| **OF TG NR** | 4 | 37 | 913 | 199 |

**Supplementary Table 2.- Mean ± SEM of analyzed mitochondrial parameters for each experimental group.** AM: adult males; AF: adult females; OM: old males; OF: old females. WT: wild type; TG: transgenic; CT: control diet; NR: nicotinamide riboside supplemented diet.

|  | **Mitochondrial area** | **Mitochondrial circularity** | **Relative mitochondrial abundance** | **Relative mitochondrial mass** |
| --- | --- | --- | --- | --- |
| **Group** | **Mean ± SEM** | **Mean ± SEM** | **Mean ± SEM** | **Mean ± SEM** |
| **AM WT CT** | 0.370 ± 0.018 | 0.756 ± 0.011 | 0.499 ± 0.028 | 0.174 ± 0.008 |
| **AM TG CT** | 0.348 ± 0.016 | 0.773 ± 0.007 | 0.550 ± 0.022 | 0.175 ± 0.008 |
| **AM WT NR** | 0.333 ± 0.018 | 0.751 ± 0.009 | 0.455 ± 0.032 | 0.161 ± 0.015 |
| **AM TG NR** | 0.351 ± 0.032 | 0.760 ± 0.010 | 0.674 ± 0.042 | 0.208 ± 0.014 |
| **OM WT CT** | 0.475 ± 0.035 | 0.743 ± 0.020 | 0.429 ± 0.029 | 0.200 ± 0.021 |
| **OM TG CT** | 0.429 ± 0.029 | 0.762 ± 0.014 | 0.392 ± 0.031 | 0.168 ± 0.015 |
| **OM WT NR** | 0.355 ± 0.031 | 0.780 ± 0.011 | 0.511 ± 0.055 | 0.150 ± 0.016 |
| **OM TG NR** | 0.339 ± 0.018 | 0.736 ± 0.013 | 0.445 ± 0.029 | 0.158 ± 0.016 |
| **AF WT CT** | 0.449 ± 0.040 | 0.743 ± 0.010 | 0.619 ± 0.039 | 0.190 ± 0.011 |
| **AF TG CT** | 0.362 ± 0.020 | 0.771 ± 0.013 | 0.507 ± 0.023 | 0.175 ± 0.012 |
| **AF WT NR** | 0.314 ± 0.016 | 0.783 ± 0.007 | 0.496 ± 0.030 | 0.165 ± 0.011 |
| **AF TG NR** | 0.424 ± 0.030 | 0.769 ± 0.008 | 0.527 ± 0.032 | 0.208 ± 0.010 |
| **OF WT CT** | 0.288 ± 0.020 | 0.776 ± 0.013 | 0.518 ± 0.035 | 0.143 ± 0.010 |
| **OF TG CT** | 0.436 ± 0.045 | 0.742 ± 0.008 | 0.416 ± 0.032 | 0.158 ± 0.011 |
| **OF WT NR** | 0.335 ± 0.024 | 0.780 ± 0.011 | 0.430 ± 0.026 | 0.140 ± 0.011 |
| **OF TG NR** | 0.368 ± 0.035 | 0.760 ± 0.011 | 0.407 ± 0.018 | 0.146 ± 0.013 |

**Supplementary Table 3.- Mean ± SEM of analyzed MERCS parameters for each experimental group.** AM: adult males; AF: adult females; OM: old males; OF: old females. WT: wild type; TG: transgenic; CT: control diet; NR: nicotinamide riboside supplemented diet.

|  | **Relative MERCS abundance** | **MERCS distance** | **MERCS length** |
| --- | --- | --- | --- |
| **Group** | **Mean ± SEM** | **Mean ± SEM** | **Mean ± SEM** |
| **AM WT CT** | 0.107 ± 0.013 | 42.42 ± 1.990 | 190.6 ± 15.550 |
| **AM TG CT** | 0.151 ± 0.011 | 27.35 ± 0.783 | 141.7 ± 8.010 |
| **AM WT NR** | 0.196 ± 0.014 | 32.31 ± 1.278 | 171.9 ± 8.610 |
| **AM TG NR** | 0.227 ± 0.192 | 27.27 ± 1.057 | 140.3 ± 9.930 |
| **OM WT CT** | 0.080 ± 0.006 | 39.50 ± 2.061 | 166.9 ± 13.67 |
| **OM TG CT** | 0.087 ± 0.013 | 38.06 ± 2.840 | 81.16 ± 6.438 |
| **OM WT NR** | 0.187 ± 0.023 | 34.39 ± 1.863 | 149.5 ± 12.29 |
| **OM TG NR** | 0.165 ± 0.011 | 27.83 ± 0.965 | 144.6 ± 11.92 |
| **AF WT CT** | 0.097 ± 0.010 | 39.21 ± 2.069 | 174.0 ± 12.030 |
| **AF TG CT** | 0.119 ± 0.015 | 27.97 ± 1.547 | 140.9 ± 9.616 |
| **AF WT NR** | 0.166 ± 0.013 | 28.47 ± 0.941 | 188.4 ± 14.800 |
| **AF TG NR** | 0.155 ± 0.013 | 32.22 ± 1.216 | 147.5 ± 11.100 |
| **OF WT CT** | 0.079 ± 0.008 | 28.91 ± 1.881 | 112.8 ± 12.380 |
| **OF TG CT** | 0.223 ± 0.020 | 36.95 ± 1.811 | 135.6 ± 9.320 |
| **OF WT NR** | 0.132 ± 0.010 | 33.71 ± 2.123 | 158.1 ± 11.330 |
| **OF TG NR** | 0.165 ± 0.015 | 36.55 ± 1.518 | 141.4 ± 12.410 |

**SUPPLEMENTARY FIGURE LEGENDS**

**Supplementary Figure 1.- STRING output from differential expressed DCT-related genes.** A: network generated using STRING platform. B: enriched GO terms and color code.

**Supplementary Figure 2.- Mitochondria-endoplasmic reticulum contact sites (MERCS) distance and length in distal convoluted tubule cells under the different experimental conditions.** Supplementary Figure 2A shows MERCS distance. Suppl. Fig. 2B shows MERCS length. Measurements are expressed nanometers (nm). See Figure 5 caption for a description of the panel arrangement. t: tendency (p < 0.1); *: p < 0.05; **: p < 0.01; ***: p < 0.001; ****: p < 0.0001. In Supp. Fig. 2A, a***: p < 0.001 vs AM TG CT; b**: p < 0.01 vs AF WT CT; c**: p < 0.01 vs AF TG CT; d**: p < 0.01 vs OM WT CT. In Supp. Fig. 2B, a*: p < 0.05 vs AM TG CT; b*: p < 0.05 vs AF WT CT; c*: p < 0.05 vs OM WT CT.

**SUPPLEMENTARY FIGURE 1**

**
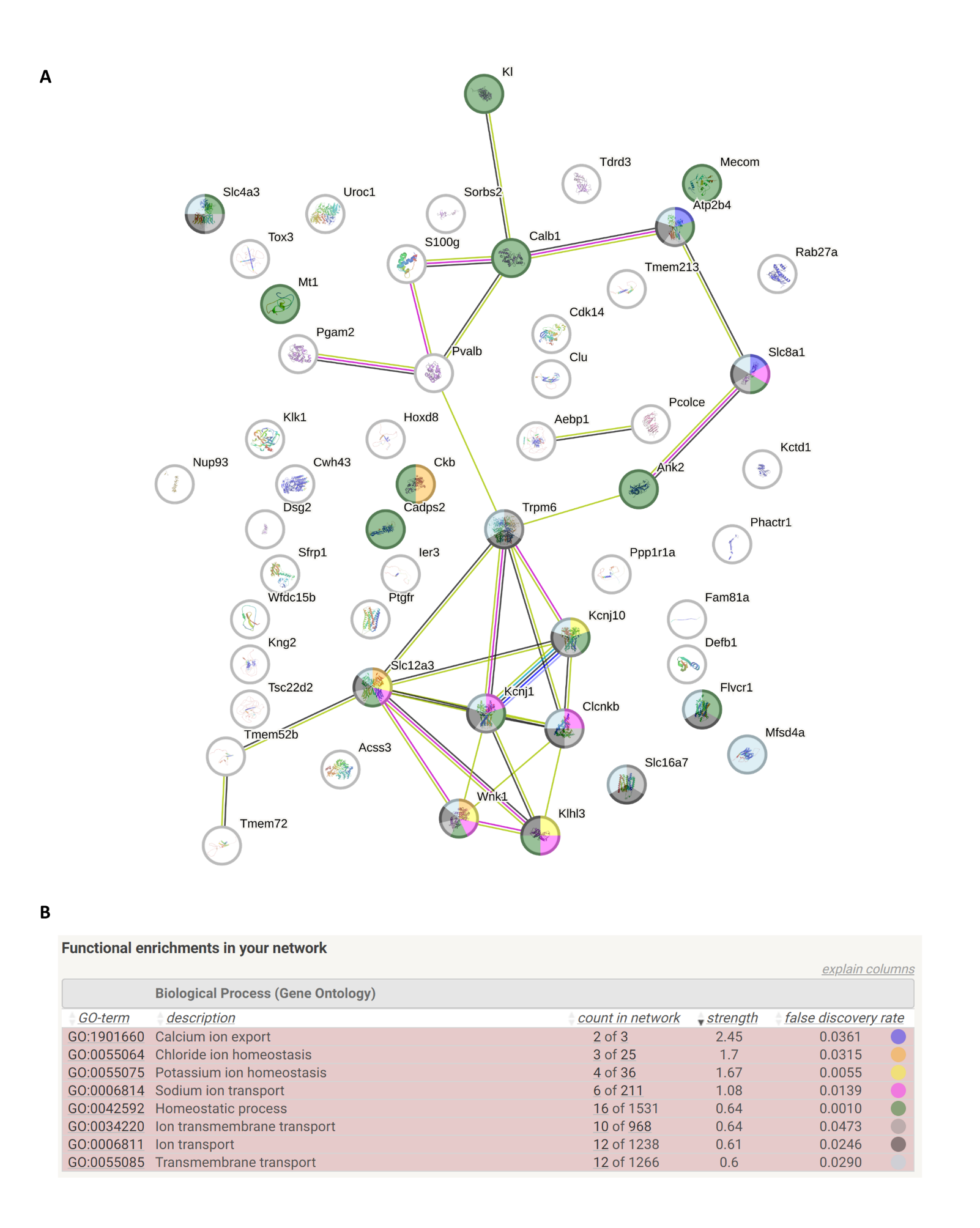
**

**SUPPLEMENTARY FIGURE 2**

**
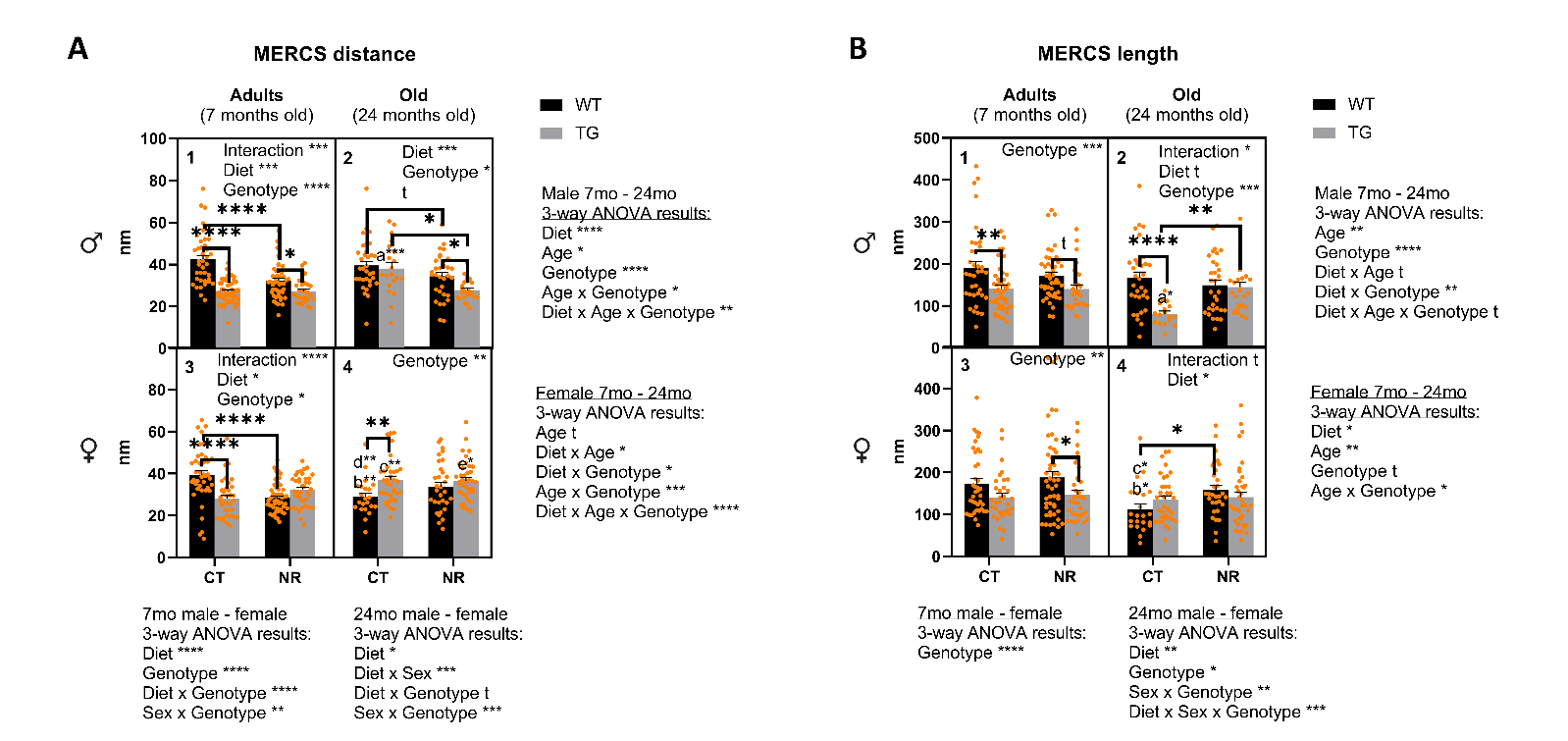
**
